# Psoriasis and Metabolic Syndrome – scientific evidence and therapeutic implications

**Published:** 2014

**Authors:** VM Voiculescu, M Lupu, L Papagheorghe, C Giurcaneanu, E Micu

**Affiliations:** * ”Carol Davila” University of Medicine and Pharmacy, Bucharest, Romania; ** “Elias” University Emergency Hospital, Bucharest, Romania; *** Humanitas Clinic, Bucharest, Romania

**Keywords:** psoriasis, metabolic syndrome, inflammation

## Abstract

Psoriasis is a chronic inflammatory disease, predominantly affecting the skin, being included in the group of Immune Mediated Inflammatory Diseases. Growing evidence from the last 10 years suggests that several systemic conditions like metabolic syndrome, cardiovascular disease, diabetes, psychological disorders or inflammatory bowel disease are prevalent in psoriasis patients. The linker might be the chronic secretion of pro-inflammatory cytokines. In this current review, the scientific evidence that explains the relationship between psoriasis and the metabolic syndrome in particular will be addressed, as the metabolic syndrome comprises a group of risk factors for cardiovascular disease, thus offering an overall picture of the systemic involvement in psoriasis. An integrated approach, with an early detection and treatment of the components of the metabolic syndrome, are important steps in psoriasis management. Attention should be paid on influence of psoriasis treatment upon comorbidities and vice-versa.

## Introduction

Psoriasis is a chronic inflammatory skin disorder, immunologically mediated (Th-1, Th-17, and Th-22 activation and expansion, controlled by dendritic antigen-presenting cells). Its prevalence ranges from 0.91 in the US to 8.5 in Norway [**1**], affecting individuals of all ages, but more prevalent in first decades of life and with health-related quality-of-life reduced even in less severely affected individuals. In the last years, an important progress has been made towards the identification of the inflammatory pathways involved in psoriasis pathogenesis. A series of inflammatory molecules are produced in psoriasis skin lesions (TNF, Il-1, Il-6, Il-8, IL-17, IL-22, Il-23, vascular endothelial growth factor (VEGF), interferon-γ, etc.) [**2**] and it seems that these molecules are released in the systemic circulation based on the severity and extension of the skin lesions [**3**,**4**]. Moreover, experimental and epidemiological studies have linked certain interleukins, cytokines and hormones (adipokines) with cardiovascular disease (CVD), metabolic syndrome (MetS), obesity and diabetes, making psoriasis a risk factor in developing systemic comorbidities [**2**,**5**,**6**]. Beside inflammation, other factors have to be considered to explain these associations, like shared risk factors (i.e. smoking, alcohol consumption), treatment (i.e. immunosuppressive agents, drugs that alter lipid profile etc.) or shared genetic risk loci.

In the following sections, the relationship between MetS and psoriasis will be addressed, focusing on interactions between adipose tissue and skin, as a consequence of psoriasis activity.

**Metabolic syndrome**

There are a number of definitions and criteria used to identify MetS. The National Cholesterol Education Program’s Adult Treatment Panel III (NCEP ATP III) revised criteria from 2005 [**7**,**8**], states that MetS diagnosis is based on the presence of at least three of the following five conditions, as defined by 1) fasting glucose 100 mg/dL or greater (or receiving drug therapy for hyperglycemia); 2) blood pressure 130/85 mmHg or higher (or receiving drug therapy for hypertension); 3) triglycerides 150 mg/dL or higher (or receiving drug therapy for hypertriglyceridemia); 4) HDL cholesterol (high density lipoprotein cholesterol) less than 40 mg/dL in men or less than 50 mg/dL in women (or receiving drug therapy for reduced HDL-C); and 5) waist circumference 102 cm (40 inches) or greater in men or 88 cm (35 inches) or greater in women; in Asian American, 90 cm (35 inches) or greater in men or 80 cm (32 inches) or greater in women. The main difference between NCEP ATP III definition and International Diabetes Federation (IDF) definition [**9**] is that the latter uses central obesity as a mandatory component, plus any two of the other four components described above. It is well known that MetS increases the risk of CVD, diabetes and cancer; however, most of the published reports indicate that the syndrome does not predict cardiovascular events or disease progression any better than the sum of its components [**8**].

MetS is associated with systemic inflammation, with elevated pro-inflammatory cytokines like TNF-α. However, the relationship with chronic inflammatory diseases is poorly understood, although rapidly growing evidence is emerging in the last years to fulfill this gap.

**Metabolic syndrome and psoriasis**

A recent meta-analysis [**10**] found that psoriasis patients have higher prevalence of MetS compared with the general population, and patients with more severe psoriasis have greater odds of MetS than those with milder psoriasis. Armstrong et al. [**10**] found that most of the studies reported a high prevalence of MetS in psoriasis patients, ranging from 14 to 40%, suggesting that MetS is a common problem for psoriasis patients.

In a large population-based study from UK, Langan et al. [**11**] found that psoriasis is associated with MetS in a ‘‘dose–response’’ manner, with a 22% increase in the odds of developing the MetS in those with mild psoriasis, 56% increase in those with moderate disease, and a 98% increase in those with severe psoriasis. Next, the scientific evidence so far, concerning interrelationships between inflammation/psoriasis and each of the components of MetS, will be briefly summarized.

**Insulin resistance and diabetes mellitus**

Insulin resistance is a condition in which normal amounts of insulin are inadequate to produce normal insulin response from fat, muscles and liver cells; it is a state that precedes type 2 diabetes mellitus. Until now, insulin resistance has explained most if not all of the components of MetS [**8**]. It seems that inflammatory mediators may be involved in insulin resistance, such as TNF-α, Il-6, leptin, adiponectin [**12**,**13**], mediators that are also disturbed in psoriasis (further detailed below).

Insulin resistance was found in non-obese patients with psoriasis [**14**] and insulin resistance was correlated with psoriasis index severity area [**15**]. Several studies have found an increased risk of diabetes in psoriasis patients [**16**,**17**]. Thus, it seems that the risk of diabetes in psoriasis is linked to insulin resistance [**18**].

**Hypertension**

Psoriasis patients have an alteration of the renin-angiotensin-aldosterone system (RAAS), with elevated plasma renin activity and elevated angiotensin-converting enzyme activity [**19**,**20**]. Several studies, including a recent meta-analysis, have shown an increased prevalence of hypertension among psoriasis patients [**21**,**22**].

Adipose tissue is an important source of angiotensinogen, the precursor of angiotensin, which plays a major role in blood pressure control. Angiotensinogen microRNA expression is increased in visceral fat. Moreover, it is known that weight loss leads to blood pressure reduction. Beside angiotensinogen, resistin and leptin secretion from adipose tissue have also been implicated in hypertension from MetS [**23**]. All these evidence provide an explanation of the relationship between hypertension and obesity in the MetS.

The RAAS may induce osteopontin expression [**24**], a pro-inflammatory cytokine, and reduce adiponectin expression [**25**], an anti-inflammatory cytokine. These cytokines are shown to be abnormal when expressed in psoriasis: increased osteopontin and decreased adiponectin levels, significantly associated with the severity of psoriasis and MetS [**26**].

**Dyslipidemia**

Dyslipidemia is a widely accepted term that comprises any plasma lipid abnormality, usually defined as increased low-density lipoprotein, very low-density lipoprotein, and triglyceride levels and decreased high-density lipoprotein levels. A recent large systematic review [**27**] found that psoriasis patients have a higher incidence of dyslipidemia than the general population.

In vitro evidence shows a common inflammatory background between psoriasis and dyslipidemia. Il-1, Il-6 and TNF-α that mediate psoriasis may alter the hepatocyte function and the arterial smooth muscle cells, which will finally lead to arterial plaque development [**28**]. Moreover, these interleukins increase lipid levels [**29**]. Psoriasis is associated with increased oxidative stress [**30**,**31**], as most human diseases are, and oxidized-LDL is found to be elevated in severe psoriasis [**32**].

**Obesity**

Obesity is associated with a chronic low level of inflammation [**33**]. Central obesity plays a central role in MetS development and seems to precede the appearance of all the other components of MetS [**34**].

Activated macrophages in the adipose tissue stimulate adipocytes to secrete adipocytokines (adipokines), such as TNF-α, IL-6, leptin, and visfatin, which may also play a role in the pathogenesis of psoriasis [**35**-**37**]. Most of the current available data refer to leptin and adiponectin [**33**]. Leptin circulating levels correlate with fat mass and leptin exerts important roles in inflammation (stimulates production of pro-inflammatory cytokines like TNF-α, and IL-6) [**33**], stimulate keratinocyte proliferation and angiogenesis [**38**]. Psoriasis patients have high levels of leptin [**39**] and these high levels may derive both from the adipose tissue in obese patients and from inflammation. Adiponectin is inversely correlated with obesity, it is an anti-inflammatory molecule, with anti-atherogenic properties [**40**] and psoriasis patients have decreased levels of adiponectin [**26**].

VEGF promotes angiogenesis and endothelial cells activation and is increased in psoriatic skin and correlates with the severity of the disease [**41**]. Increased levels of VEGF are found in hyperinsulinemic states, such as MetS, deriving from adipocytes [**42**]. Among all adipose tissues in the body, omentum expresses the highest level of VEGF [**43**]. A recent meta-analysis that comprises 16 observational studies found that psoriasis patients have a higher prevalence and incidence of obesity, compared with the general population [**21**].

**Therapeutic implications**

The current therapies for psoriasis include topical agents, phototherapies, systemic therapy (immunosuppressive drugs) and biotherapies (biologics). Some of the drugs used for the psoriasis treatment have been shown to modify risk components of MetS while other exacerbate them. Obesity is a risk factor for MetS and weight loss may improve psoriasis [**35**]. A recent controlled study showed that weight loss improved the therapeutic response to a low dose of cyclosporine in obese patients with moderate-to severe chronic plaque psoriasis [**44**]. Patients on cyclosporine should monitor their blood pressure.

Some of the anti-TNF biologics are shown to reduce C-reactive protein levels [****45****], reduce lipid peroxidation and increase high-density lipoprotein levels [**46**], thus having a beneficial systemic effect. Dermatologists should be aware that patients with psoriasis both on anti-TNF therapies and insulin for diabetes may experience hypoglycemia [**47**].

Other drugs used for psoriasis have negative effects like the alteration of lipids profile in the case of retinoids and cyclosporine, while some drugs used for MetS like fibrates or even statins may exacerbate psoriasis [**48**, **49**]. For statins, other authors suggest beneficial effects in psoriasis [**50**], that might derive from anti-TNF properties of statins.

## Conclusions

Psoriasis is currently considered a “T-cell mediated disease”, and shares a common immune profile with MetS, both of them having increased Th type 1 proinflammatory cytokines, with a wide range of actions: on insulin signaling, lipid metabolism, adipogenesis [**2**]. These pieces of evidence may explain the increased prevalence of MetS in psoriasis patients.

Information about the degree of psoriasis severity and duration still lacks, being necessary to increase MetS risk. However, the current recommendation is that all psoriasis patients should undergo a complete evaluation and treatment for all the components of MetS and physicians should be aware of the potential increased risk of psoriasis, in particular moderate to severe forms, for MetS and thus CVD and increased mortality risk.

**Sources of funding:** None

**Disclosures:** None
